# Copolymer Involving 2-Hydroxyethyl Methacrylate and 2-Chloroquinyl Methacrylate: Synthesis, Characterization and In Vitro 2-Hydroxychloroquine Delivery Application

**DOI:** 10.3390/polym13234072

**Published:** 2021-11-18

**Authors:** Abeer Aljubailah, Wafa Nazzal Odis Alharbi, Ahmed S. Haidyrah, Tahani Saad Al-Garni, Waseem Sharaf Saeed, Abdelhabib Semlali, Saad M. S. Alqahtani, Ahmad Abdulaziz Al-Owais, Abdulnasser Mahmoud Karami, Taieb Aouak

**Affiliations:** 1 Department of Chemistry, College of Science, Imam Mohammad Ibn Saud Islamic University (IMSIU), Riyadh 13623, Saudi Arabia; akaljubailah@imamu.edu.sa (A.A.); wnalharbi@imamu.edu.sa (W.N.O.A.); 2 Nuclear and Radiological Control Unit, King Abdulaziz City for Science and Technology (KACST), Riyadh 11442, Saudi Arabia; ahydrah@kacst.edu.sa; 3 Chemistry Department, College of Science, King Saud University, Riyadh 11451, Saudi Arabia; tahanis@ksu.edu.sa (T.S.A.-G.); salqahtani2@ksu.edu.sa (S.M.S.A.); aowais@ksu.edu.sa (A.A.A.-O.); akarami@ksu.edu.sa (A.M.K.); 4 Engineer Abdullah Bugshan Research Chair for Dental and Oral Rehabilitation, College of Dentistry, King Saud University, Riyadh 11545, Saudi Arabia; 5 Groupe de Recherche en Écologie Buccale, Faculté de Médecin Dentaire, Université Laval, Quebec City, QC G1V 0A6, Canada; abdelhabib.semlali@greb.ulaval.ca

**Keywords:** preparation and characterization, poly(2-chloroquinyl methacrylate-*co*-2-hydroxyethyl methacrylate), 2-chloroquinyl methacrylate, drug carrier system, in vitro 2-hydroxychloroquine release

## Abstract

The Poly(2-chloroquinyl methacrylate-*co*-2-hydroxyethyl methacrylate) (CQMA-*co*-HEMA) drug carrier system was prepared with different compositions through a free-radical copolymerization route involving 2-chloroquinyl methacrylate (CQMA) and 2-hydroxyethyl methacrylate) (HEMA) using azobisisobutyronitrile as the initiator. 2-Chloroquinyl methacrylate monomer (CQMA) was synthesized from 2-hydroxychloroquine (HCQ) and methacryloyl chloride by an esterification reaction using triethylenetetramine as the catalyst. The structure of the CQMA and CQMA-*co*-HEMA copolymers was confirmed by a CHN elementary analysis, Fourier transform infra-red (FTIR) and nuclear magnetic resonance (NMR) analysis. The absence of residual aggregates of HCQ or HCQMA particles in the copolymers prepared was confirmed by a differential scanning calorimeter (DSC) and XR-diffraction (XRD) analyses. The gingival epithelial cancer cell line (Ca9-22) toxicity examined by a lactate dehydrogenase (LDH) assay revealed that the grafting of HCQ onto PHEMA slightly affected (4.2–9.5%) the viability of the polymer carrier. The cell adhesion and growth on the CQMA-*co*-HEMA drug carrier specimens carried out by the (3-(4,5-dimethylthiazol-2-yl)-2,5-diphenyltetrazolium bromide) (MTT) assay revealed the best performance with the specimen containing 3.96 wt% HCQ. The diffusion of HCQ through the polymer matrix obeyed the Fickian model. The solubility of HCQ in different media was improved, in which more than 5.22 times of the solubility of HCQ powder in water was obtained. According to Belzer, the in vitro HCQ dynamic release revealed the best performance with the drug carrier system containing 4.70 wt% CQMA.

## 1. Introduction

2-Hydroxychloroquine (HCQ), also called “Plaquenil”, is a weak basic drug belonging to the family of 4-aminoquinolines. This medication is an antimalarial widely used in the treatment of systemic lupus erythematosus, rheumatoid arthritis, malaria and other autoimmune diseases [1,2]. It is also recommended to take 2-hydroxychloroquine with food to reduce stomach irritation. According to Hedya et al. [3], the addition of HCQ to cytotoxic or antiangiogenic agents can dramatically improve antitumor activity. Many tests [4,5] combining different anticancer therapies, including chemo and radiation therapies with HCQ, have shown very satisfactory results. This drug is mainly found in a dicationic form in physiological pH media and is readily trapped in cellular tissues resulting in a tissue deposition effect. According to a report published in 2005 by Day et al. [6], the distribution of HCQ in the bloodstream is relatively slow, which delays the onset of an antirheumatic effect. Therefore, a high dose of HCQ is needed to reach a steady state more quickly. According to Ono et al. [7], a dose of 400 to 600 mg is usually prescribed for normal men, while 200 to 400 mg is the maintenance dose. A higher dose results in a greater magnitude of dose-dependent side effects such as retinopathy, which is mainly due to a build-up of threads in the cornea [8]. Antirheumatic drugs, of which HCQ is a part of, can cause serious gastrointestinal complications that become more acute at higher doses. As a result, symptomatic treatments with glucocorticoids and non-steroidal anti-rheumatic drugs (NSAIDs) are known to induce gastric or duodenal ulcers, especially in association with combination therapy [9]. The control of the HCQ amount released during gastrointestinal transit (GIT) in the different organs is necessary in order to minimize the release of HCQ in organs sensitive to unwanted side effects and, more particularly, in the stomach.

In the absence of an established treatment regimen, many drug reuse strategies have been emerging to treat corona virus disease (COVID-19) [10]. Indeed, among these drugs, HCQ in combination with azithromycin has been recommended to treat this virus, especially in older patients or patients with underlying conditions and severe symptoms [11,12]. Despite the promise of the reuse of this drug, there are concerns about its toxicity, because some in vitro studies suggest that the dose needed to be effective against COVID-19 may be higher than that used in malaria. The World Health Organization (WHO) is currently conducting clinical tests (SOLIDARITY) to assess the effectiveness of 2-hydroxychloroquine as a treatment for COVID-19, while some countries have already included treatment with 2-hydroxychloroquine in their clinical advice for patients with COVID-19 [13,14]. In addition, the HCQ base has a partition coefficient ranged between 2.89 and 3.87 and a water solubility of 26.1 mg·L^−1^ [15]. These two properties place this drug in the Biopharmaceutical Classification System-II (BCS-II); in other words; this medication is highly permeable but poorly soluble. In its sodium sulfate form, HCQ has excellent bioavailability with an average fraction of 0.74 absorbed doses [16]. On the other hand, this salt is highly soluble in gastric fluid but can recrystallize in the environment at a higher pH (small intestine), because the mother base is highly insoluble in this medium. In addition, an increase in the pH of the stomach in the postprandial state can also present a solubility challenge. In both cases, a decrease in the solubility of the drug is conceivable, which results in a lower in vivo exposure and, as such, an approach allowing the solubility can overcome this variation in solubility during gastrointestinal transit (GIT). In particular, and as recently described in the literature by Zhang et al., salt forms of drugs can experience a significant decrease in solubility upon transition to the higher pH of the small intestine [17].

Poly(2-hydroxyethyl methacrylate) (PHEMA) has exhibited very interesting properties in its application in the biomedical field due to its high water content, its non-toxicity and its favorable biocompatibility. This polymer is easily synthesized by free-radical polymerization involving 2-hydroethyl methacrylate as the monomer and azobisisobutyronitile (AIBN) as the initiator. The ease of handling by the formulation chemistry has allowed this polymer a wide application in the biomedical field, such as contact lenses [18,19], keratoprostheses and as orbital implants [20]. The presence of the hydroxyl and carboxyl groups on the substituent of this polymer give it compatibility with water, a hydrolytic stability, support for mechanical resistance [21] and a better adhesion of cells [22,23].

In this work, to increase the solubility of the HCQ base in pH-neutral media (intestines) and reduce the amount of HCQ released in acidic media (stomach), a new monomer (CQMA) involving HCQ and methacryloyl chloride was synthesized via a catalytic esterification reaction. The new monomer obtained was copolymerized at different ratios with 2-hydroxyethylmethacrylate (HEMA) using the radical polymerization route to obtain poly(2-chloroquinylmethacrylate-*co*-2-hydroxyethylmethacrylate) (CQMA-*co*-HEMA). The structures of monomers and copolymers obtained were characterized by the Fourier transform infrared (FTIR), nuclear magnetic resonance (NMR) and CHN elementary analyses. The absence of free drug particles aggregated incrusted in the copolymer matrix was confirmed by DSC and XRD methods. The cell toxicity was examined by the lactate dehydrogenase (LDH) assay on the gingival epithelial cancer cell line (Ca9-22) and the cell adhesion and growth on the CQMA-*co*-HEMA drug carrier was examined by the 3-(4,5-dimethylthiazol-2-yl)-2,5-diphenyltetrazolium bromide (MTT) test. The in vitro dynamic release of the HCQ base from these drug carrier systems occur in different pH media through a retroesterification reaction, in which the influence of the swelling capacity of the drug carrier system, the drug content grafting in the copolymer and pH media are investigated.

## 2. Materials and Methods

### 2.1. Chemicals

HEMA (purity 98%), triethylenetetramine (purity > 97%), methacryloyl chloride (purity ≥ 97%), AIBN (purity 99%) and chloroform (purity ≥ 99%) were supplied from Sigma-Aldrich (Taufkirchen, Germany). Plaquenil commercial trade tablets were manufactured and commercialized by Aventis Pharma Limited (Guildford, UK), Sanofi (Paris, France), Kyowa Hakko (Galashiels, UK). All the chemicals were used without further purification except the monomer which was purified by distillation under reduced pressure.

### 2.2. Extraction of 2-Hydroxychloroquine from the Plaquenil Commercial Tablets

A determined amount of 200 mg of Plaquenil tablets was finely ground using a quartz mortar, then added in small portions with continuous stirring in distilled water until complete dissolution (~72 h). Two drops of phenolphthalein indicator were added to the aqueous solution, then titrated by 2% by volume ammonia solution until pink color persisted. In order to extract HCQ from the aqueous solution, the obtained solution was then transferred in a separator funnel containing equivalent amount of diethyl ether. HCQ was extracted from the organic layer by vaporization of diethyl ether using a rotary evaporator. The purity of HCQ was confirmed by NMR analysis. The physicochemical characteristics of this molecule are gathered in Table 1.

### 2.3. Synthesis of 2-Chloroquinyl Methacrylate (CQMA)

**Scheme 1:**
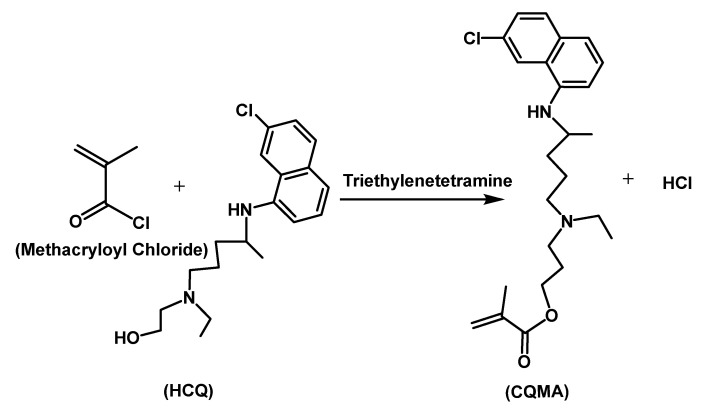
Synthesis reaction of 2-chloroquinyl methacrylate.

CQMA was synthesized by an esterification reaction involving HCQ and methacryloyl chloride using triethylenetetramine as a catalyst according to the reaction in Scheme 1. In a 250 mL two-necked flask, 10 g of HCQ was dissolved by stirring in 100 mL of chloroform, and then triethylenetetramine was added to this mixture so that the 2-hydroxychloroquine/triethylenetetramine molar ratio was 1:3 in order to ensure complete consumption of HCQ during the esterification reaction. A reflux condenser was connected through the main opening of the flask containing the HCQ/triethylenetetramine mixture in chloroform, through which a stream of nitrogen gas at low flow (3 mL·min^−1^) passed. The whole was placed in an ice bath for 10 min. The excess of methacryloyl chloride diluted in chloroform was then added dropwise with moderate stirring to the preceding solution using an addition bulb. The reaction took place in a dark atmosphere under a stream of nitrogen and under reflux during the whole period of the reaction. CQMA was isolated from the reaction mixture by a complete evaporation of solvent and liquid residual reactants using rotary system. The residual HCQ was removed by washing the CQMA obtained three times by an excess of distillated water, then dried in a vacuum oven until constant mass.

### 2.4. Preparation of CQMA-co-HEMA Drug Carrier Systems

**Scheme 2:**
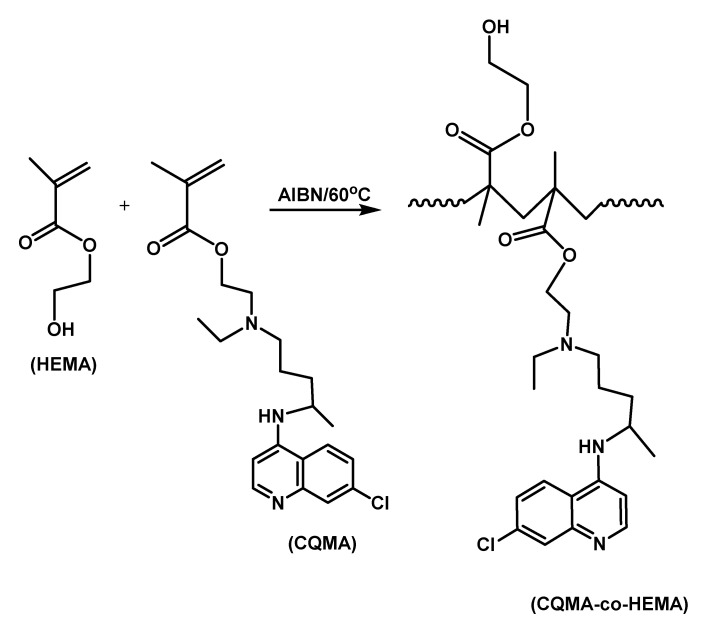
Free-radical copolymerization of CQMA with HEMA.

CQMA was copolymerized with HEMA at 60 °C through a free-radical polymerization route under nitrogen gas using AIBN as initiator (Scheme 2). Different copolymers containing different co-monomer ratios were synthesized and the preparation conditions are summarized in Table 2.

### 2.5. Characterization

The new synthesized monomer and copolymers were characterized by different techniques. The FTIR spectra of the samples were recorded on a Nicolet iS10 spectrometer (Thermo Scientific, Madison, WI, USA), equipped with an attenuated total reflection (ATR; diamond crystal) accessory. Spectra were obtained over a region of 4000–500 cm^−1^ at room temperature and acquired with a total of 32 scans per spectrum and resolution of 2 cm^−1^. The ^1^H NMR and ^13^C NMR spectra of samples were taken at 400 and 100 MHz, respectively, on a spectrometer (JEOL Resonance, JEOL, Tokyo, Japan) using deuterated dimethyl sulfoxide (DMSO-d_6_) as a solvent. The DSC thermograms were traced by a Shimadzu DSC 60A (Kyoto, Japan) system previously calibrated with indium. In total, 8–10 mg of monomer and copolymer powders was packed in aluminum DSC pans before being placed in a DSC cell, then heated under nitrogen gas from 30 to 200 °C at a heating rate of 20 °C·min^−1^. Data were collected from the second scan run for all samples. No degradation phenomena of HCQ, CQMA and CQMA-*co*-HEMA samples were observed in all DSC thermograms in the investigated temperature range, noting that the *T*_g_ value was estimated as the midpoint of the heat capacity change with temperature and the *T*_m_ at the top of the melting peak. X-ray diffraction of pure HCQ and CQMA microparticles and copolymers was recorded by (Rigaku D_max_ 2000, The Woodlands, TX, USA) diffractometer using an anode tube of Cu working with voltage of 40 KV and a generator current of 100 mA.

The range of diffraction angle was 0–80 two theta. The samples were used as thin films, except that of pure HCQ and prepared CQMA which were analyzed as powder. SEM images of film samples before and after the release process were performed on a JEOL JSM-6610LV scanning electron microscope (SEM) (Tokyo, Japan) at an accelerating voltage of 10 kV. The surface and cross-sections of samples were first sputter-coated with a thin layer of gold and then observed at magnification range of 300–3000×.

### 2.6. Toxicity and Cell Adhesion

#### 2.6.1. Cell Culture Conditions

The gingival epithelial cancer cell line (Ca9-22) was obtained from RIKEN BioResource Research Center, Tsukuba, Japan. Ca9-22 cells were cultured in RPMI-1640 medium (Thermo Fisher Scientific, Burlington, ON, Canada), supplemented with L-glutamine, 5% fetal bovine serum (FBS) (Gibco; Thermo Fisher Scientific, Burlington, ON, Canada) and antibiotics (Sigma-Aldrich, Oakville, ON, Canada). Cell cultures were performed at 37 °C in humidified incubator with 5%; CO_2_ atmosphere conditions and cell culture medium were changed every two days.

#### 2.6.2. Cell Adhesion by MTT Assay

Each biomaterial was placed in 24-well plates and cancer gingival cells at 100 × 10^3^ cells/wells were seeded directly in biomaterial sample, then cultured for 24 h with RPMI medium. After 24 h of adhesion cells, an MTT assay was performed as described by Semlali et al. [25,26]. Briefly, each culture was supplemented with 10% of MTT (5 mg/mL) and incubated for 3 h at 37 °C. The stained cells were then lysed using 500 µL of isopropanol-HCl solution at 0.04 M with agitation for 15 min. An amount of 100 µL triplicate of lysis solution was transferred to 96-well plates to be read at 550 nm using an iMark microplate reader (Bio-Rad, Mississauga, ON, Canada). Cell adhesion levels were determined at 550 nm by means of the following formula:

(1) $$Cell\;viability\left(\%\right)=\frac{OD\left(treated\;cells\right)-OD\left(Blank\right)}{OD\left(Control\;cell\right)-OD\left(Blank\right)}\times 100$$

where *OD* is the optical density.

#### 2.6.3. Cell Toxicity by LDH Assay

Ca9-22 cells were seeded at 10^5^/well in 24-well cell culture plates for 48 h. The LDH activity was assessed in culture supernatants collected from cell adhered in different biomaterials for 48 h. LDH activity was measured using LDH (Sigma-Aldrich, Oakville, ON, Canada) [27]. Triton (1%) was used as positive control (100% of toxicity) and pure PHEMA as negative control (13% of toxicity).

### 2.7. Mass Transfer

The mass transfer of media in the polymers was carried out by gravimetric method [28]. CQMA-*co*-HEMA films of known masses and thicknesses were immersed in excess of water at 37 °C in media of pH 1, 3, 5 and 7. These samples were then removed after time intervals, wiped with tissue paper from water droplets deposited on both surfaces, then immediately weighed. Each operation lasted until the films were saturated (constant weight). Each process was triplicate and the masses of media absorbed were taken from the arithmetic average. The swelling of the film sample in weight percent was calculated according to Equation (2):

(2) $$S\left(\mathrm{wt}\%\right)=\frac{m_{t}+m_{\mathrm{HCQ}}-m_{o}}{m_{o}}\times 100$$

where $m_{t}$ and $m_{o}$ are the masses of the film sample at time *t* and *t_o_*, respectively. $m_{\mathrm{HCQ}}$ is the mass of HCQ released during time *t*.

### 2.8. In Vitro Drug Release

CQMA-*co*-HEMA films with a square surface area of 4 cm^2^ and an average thickness of 1.23 mm were suspended in 100 mL of water/hydrochloric acid solution at pH fixed at 1, 3, 5 and 7, and stirred at 100 rpm at 37 °C. Aliquots of 0.5 mL were withdrawn at time intervals and immediately replaced by water at the same pH media just after analysis. This operation kept a constant volume of media during the release process and reflected as much as possible what was actually happening in the intestines in which the HCQ released was absorbed gradually. The concentration of drug released during this period was calculated taking into account the volume of medium removed for quantitative analysis. The total mass of HCQ released during this period (*m_t_*) was calculated from its concentration deduced from the UV–visible calibration curve indicating the change in the absorbance versus the concentration of medication. It is important to note that, during the drug release process, the pH of the medium was practically not affected by the small amount of HCQ released and, hence, the addition of a buffer solution to stabilize the pH of the medium was not necessary. The percentage of HCQ released, *R* (wt%), in the media during a certain time, *t,* was calculated from Equation (3):

(3) $$R\left(\mathrm{wt}\%\right)=\frac{m_{t}-m_{o}}{m_{o}}\times 100\;$$

where $m_{o}$ is the mass of the initial HCQ incorporated in the drug carrier sample.

## 3. Results and Discussions

### 3.1. Characterization

The chemical structures of the CQMA monomer and CQMA-*co*-HEMA copolymers were confirmed by FTIR, NMR and elemental analyses. The absence of HCQ and CQMA residual particles aggregated in the copolymer samples was highlighted by DSC, XRD and SEM analyses.

#### 3.1.1. Elementary Analysis

The elemental composition of CQMA was confirmed by the CHN analysis through the consistent comparison of the experimental data with those calculated, and the results obtained are summarized in Table 3.

The composition of the copolymer in CQMA and HEMA monomeric units was also determined by this same method and the results obtained were gathered in Table 4. As these data show, the composition of these copolymers in the CQMA unit did not accurately reflect the composition in starting monomers. This seemed obvious, because the large steric hindrance of the CQMA monomer, which was much greater than that of HEMA, went in the sense of considerably reducing its reactivity with respect to HEMA.

#### 3.1.2. FTIR Analysis

**Figure 1:**
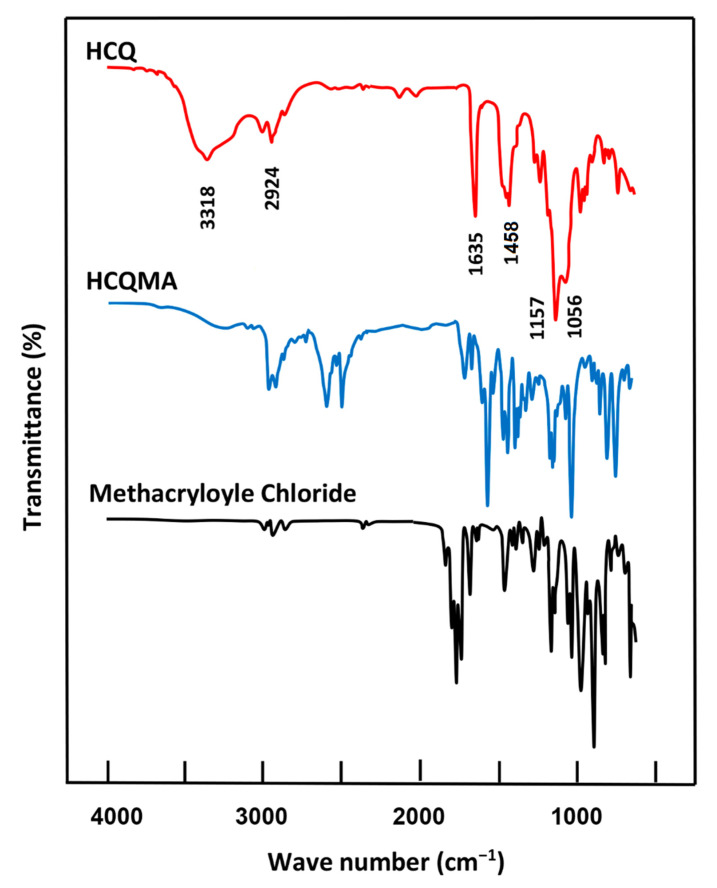
FTIR spectra of CQMA, HEMA monomers, pure HCQ and pure methacroyle chloride.

The FTIR spectrum of CQMA in Figure 1 shows the presence of the combination of the different absorption bands belonging to the two reagents HCQ and methacryloyl chloride and the total disappearance of that at 3343 cm^−1^ assigned to the hydroxyl group of the pure HCQ indicating the formation of this monomer. The CQMA spectrum confirmed the structure of this monomer through the apparition of the same absorption bands attributed to both HCQ and mathacryloyl methacrylate, except that of –OH stretching frequency which appeared in the HCQ spectrum at 3385 cm^−1^. Indeed, the main signals that appeared on the CQMA spectrum were the aromatic C–H stretching frequency at 2915 cm^−1^, aromatic C=C stretching frequency in two regions at 1634 and 1457 cm^−1^, the C–Cl stretching frequency at 1056 cm^−1^ and the C–N bending frequency at 1157 cm^−1^. The two absorption bands observed at 2500 and 2600 cm^−1^ were probably assigned to residual quaternary ammonium salts, resulting from the reaction involving methacryloyl chloride and triethylenetetramine during the monomer preparation.

**Figure 2:**
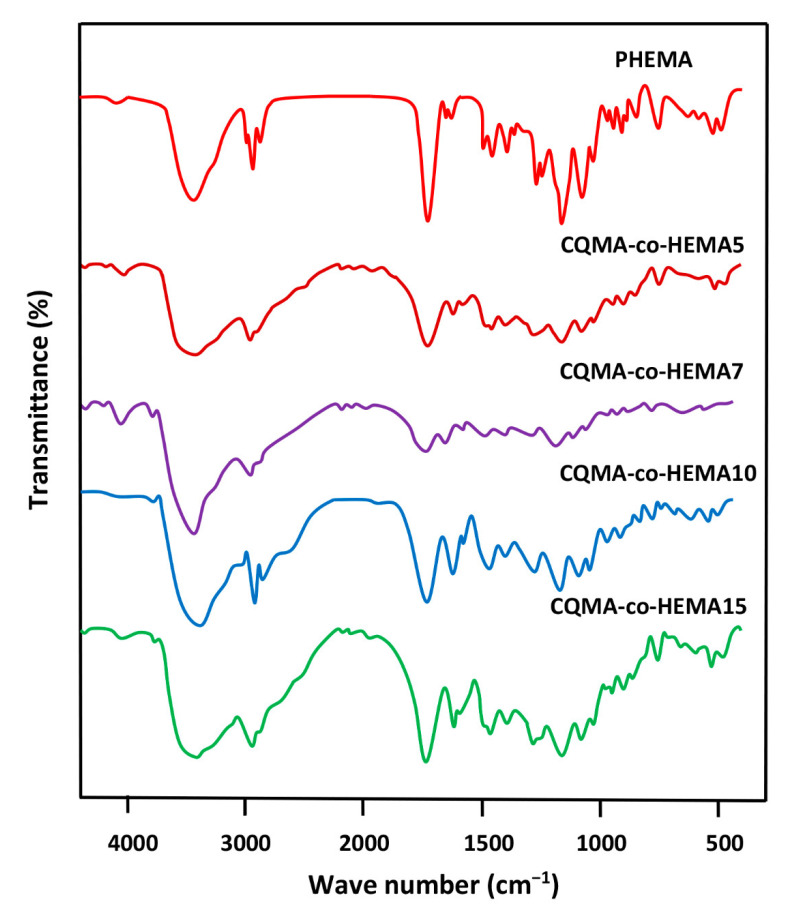
FTIR spectra of pure PHEMA, PCQMA and CQMA-*co*-HEMA copolymers.

Figure 2 shows the FTIR spectra of the CQMA-*co*-HEMA copolymer of different compositions. These spectra showed the combination of the absorption bands attributed to the two monomers HEMA and CQMA, except that which characterized the vinyl C=C double bond of the two monomers of average intensity at 1635 cm^−1^ represented in Figure 1. The bands of absorption attributed to C–Cl stretches and CN bending shown at 1057 and 1157 cm^−1^ in the CQMA monomer (Figure 1) were covered in the copolymer spectra by the –C–O–C– bands of the HEMA units, which appeared between 1321 and 1032 cm^−1^ [29].

#### 3.1.3. NMR Analysis

**Figure 3:**
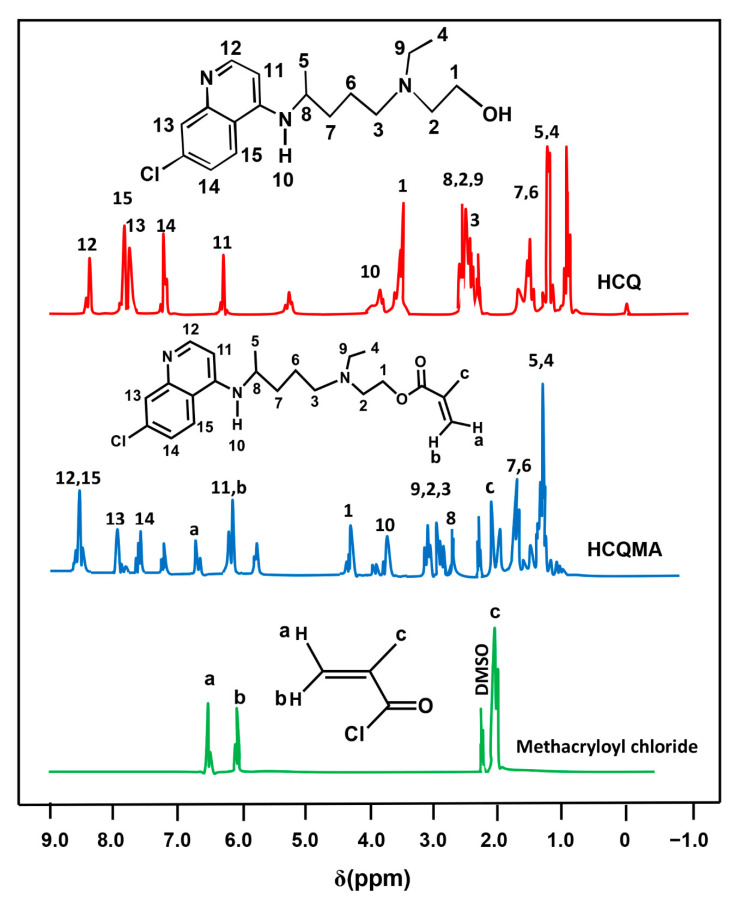
^1^H NMR spectra of pure HCQ, methacryloyl chloride and CQMA.

The ^1^H NMR spectrum of the CQMA monomer in Figure 3 presents the signals of protons involving HCQ and methacryloyl chloride with a significant shift toward the right from 3.45 to 4.32 ppm. This was due to the change in the environment of the two protons of the methylene group in position (1) belonging to HCQ to those of the ester group of the 2-chloroquinyl mathacrylate unit; thus, confirming the results of the FTIR.

**Figure 4:**
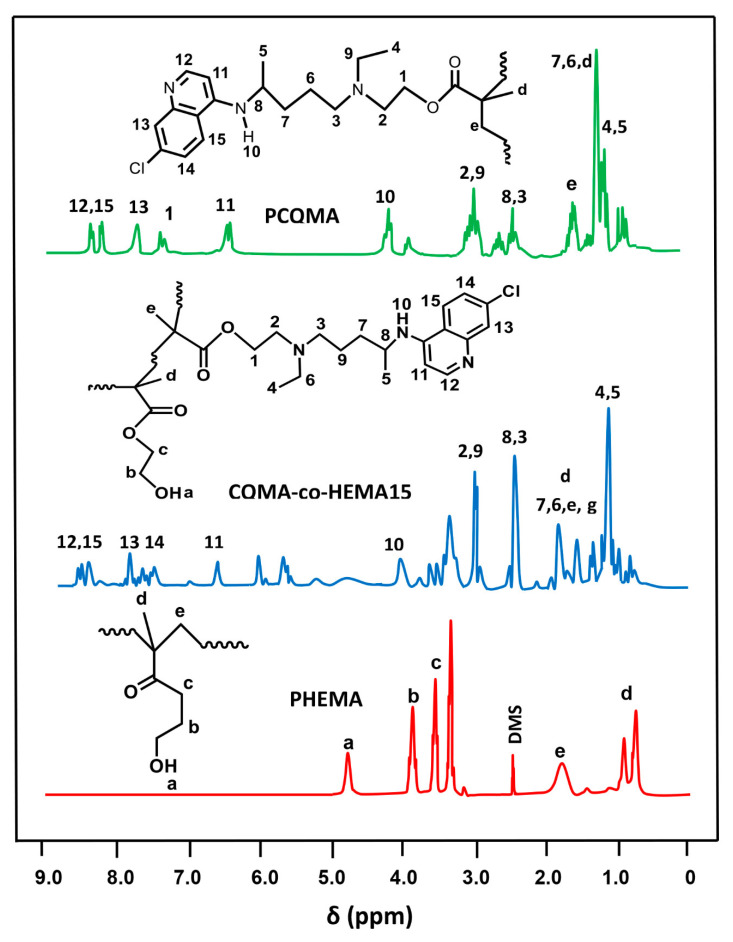
^1^H NMR spectra of PCQMA, PHEMA homopolymers and CQMA-*co*-HEMA15 copolymers.

The ^1^HNMR spectra of CQMA-*co*-HEMA15, selected among the other copolymers by their higher density in chloroquinyl groups, the PHEMA and PHCQMA homopolymers were grouped in Figure 4. As in the case of the FTIR analysis, the spectra of the copolymers revealed the presence of the combination of all the signals attributed to the two monomeric units, CQMA and HEMA, with the exception of those attributed to the protons (a) and (b) of the vinyl double bonds (sites of the polyaddition reaction), which appeared on the monomers spectra of Figure 3.

**Figure 5:**
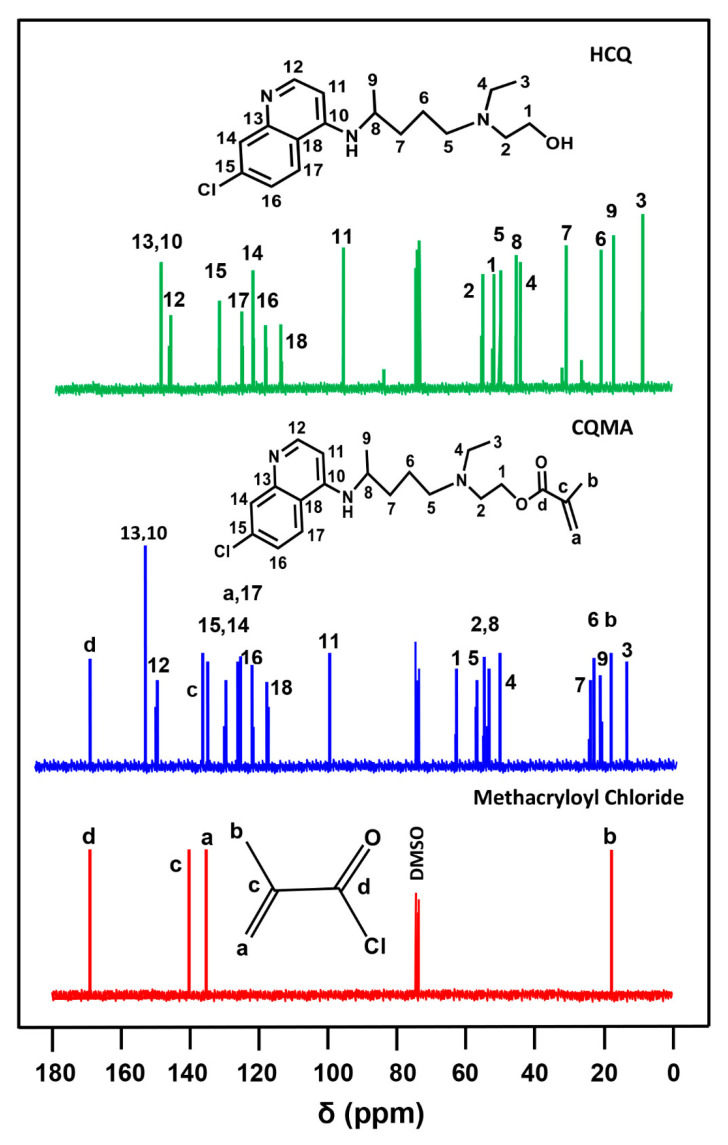
^13^C NMR spectra of pure HCQ, pure methacryloyl chloride and CQMA in DMSOd_6_.

Figure 5 shows the comparison of the ^13^C NMR spectra of the CQMA monomer with those of its reagents HCQ and methacroyl chloride. As in the case of the ^1^HNMR analysis, the structure of the CQMA monomer was demonstrated in its spectrum by the presence of all the signals attributed to the carbons of the two reagents, except that in position (1) observed at 52.4 ppm, which was directly linked to the hydroxyl group of HCQ, which shifted to the higher chemical shifts (63.5 ppm), indicating the formation of the ester group of the CQMA monomer.

**Figure 6:**
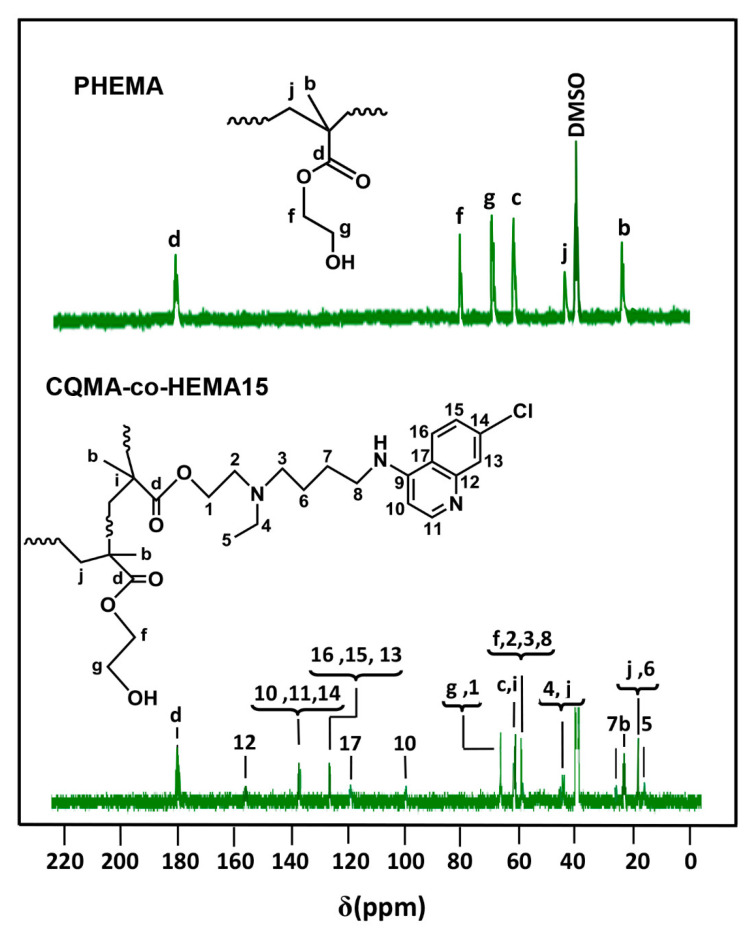
^13^C NMR spectra of PHEMA homopolymer and CQMA-*co*-HEMA15 copolymers.

The structure of the CQMA-*co*-HEMA copolymer was confirmed by the ^13^C NMR analysis from the comparison of their spectra with that of the PHEMA homopolymer. Indeed, as shown in Figure 6, the signals of carbons attributed to the CQMA and HEMA co-monomeric units were present in the spectra of the copolymer except those of the two ethylenic carbons (c) and (d) of their starting monomers (Figure 5), which were transformed into two ethenic carbons (i) and (j), respectively, during the polyaddition reaction. In addition, small shifts were observed on some signals of the carbons in the environment of sites subject to additional reactions.

#### 3.1.4. DSC Analysis

**Figure 7:**
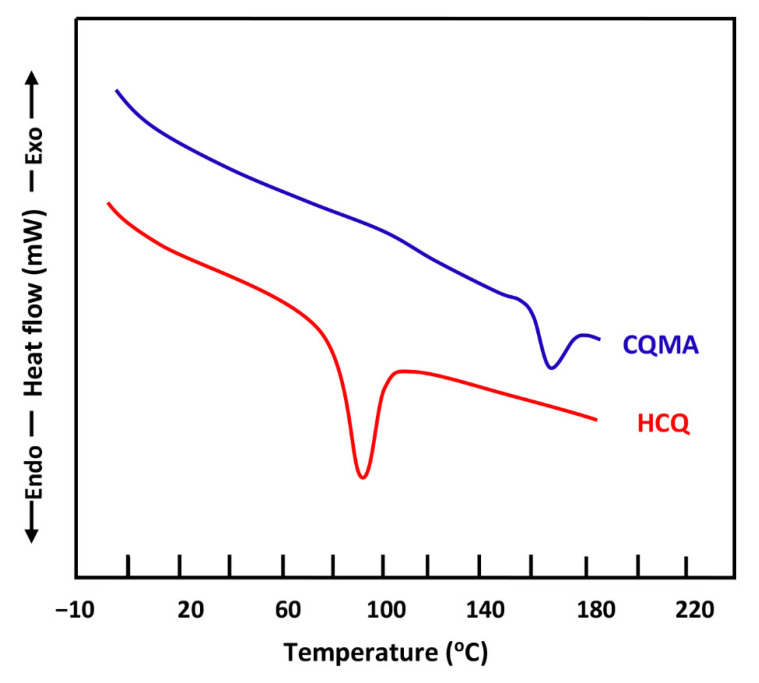
DSC thermograms of pure HCQ and CQMA monomers.

The DSC thermograms of the pure HCQ and CQMA monomers were grouped for comparison in Figure 7. The profile of each thermal curve shows a sharp endothermic peak characterizing the absorption enthalpy during the melting process. The melting temperature of each sample was taken from the top of the peak, which was 90 °C for HCQ, which agreed with the literature [15], and 167 °C for CQMA. The slight change in the heat capacity of the CQMA sample observed at 117 °C seemed to indicate a glass transition temperature, revealing the presence of a polymer resulting from a thermal polymerization of a fraction of the monomer during the DSC heating process in a nitrogen gas atmosphere.

**Figure 8:**
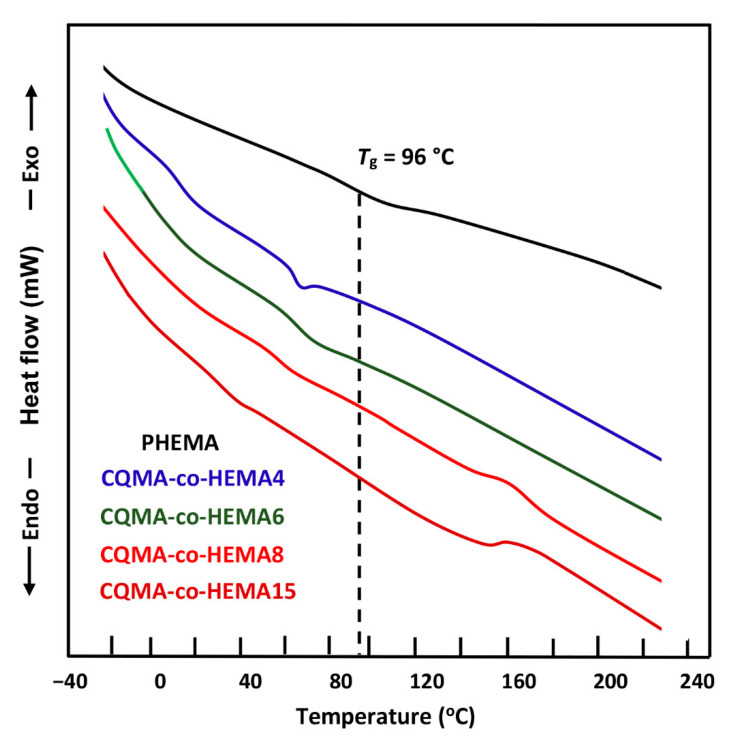
DSC thermograms of PHEMA and CQMA-*co*-HEMA with different compositions.

For CQMA-*co*-HEMA, Figure 8 shows a comparison between the thermograms of the PHEMA homopolymer and those of the copolymers involving CQMA and HEMA monomers with different compositions. As shown in these profiles, PHEMA presented a glass transition temperature, *T*_g_, at 95 °C, which agreed with that of the literature [30], while the thermal curves of the copolymer showed a shift in the *T*_g_ towards low temperatures, which increased with the CQMA content. This appeared to be evident due to the effect of the steric hindrance of the chloroquinyl methacrylyl substituent of the CQMA units. Indeed, according to Reimschuessel [31], the more the substituent of the alkyl methacrylyl units in the homopolymer is hindered, the less the values of *T*_g_s are important. The large spacing between the polymer chains caused by this substituent facilitated the sliding of the chains between them, and this reflected the reduction in *T*_g_ values. On the other hand, the more the number of these units increase in the copolymer, the less the *T*_g_ values.

#### 3.1.5. X-ray Diffraction Analysis

**Figure 9:**
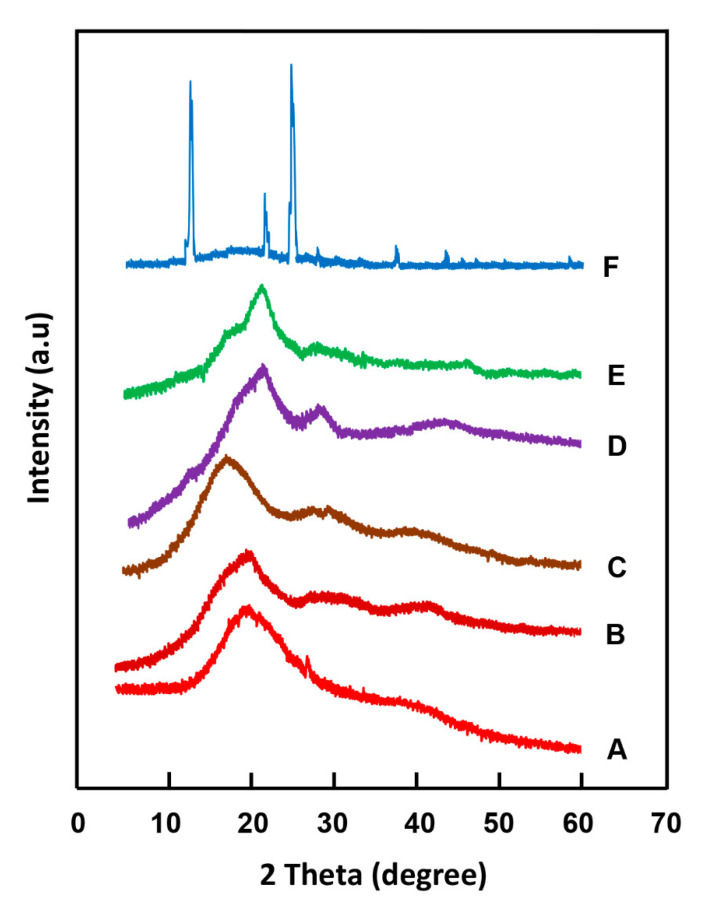
XRD patterns of: (A) PHEMA; CQMA-*co*-HEMA with different CQMA contents: (B) 5 wt%; (C) 7 wt%; (D) 10 wt%; (E) 15 wt% and (F) CQMA.

The X-ray diffractograms of the CQMA monomer, PHEMA homopolymer and CQMA-*co*-HEMA copolymers with different compositions are shown in Figure 9. The profiles of the curves attributed to the copolymers, as for the homopolymer, did not show any signs indicating a crystalline region. Indeed, the appearance of broad peaks in PHEMA centered at 19.5° 2 theta [32] and CQMA-*co*-HEMA, which slightly shifted from 20.0 to 21.2° 2 theta with the CQMA content, was due to the lack of crystallographic order of the polymeric chains; thus, leading to the formation of amorphous structures which was mainly caused by the steric hindrance of the substituent on both sides of the polymer chains. The absence of the main signals attributed to the CQMA monomer crystals observed in its spectrum at 12.3, 22.0 and 25.02 theta in the spectra of the copolymers indicated the absence of residual particles of chloroquinyl methacrylate non polymerized incrusted in the copolymer matrix; thus, confirming the results of DSC.

### 3.2. Surface Morphology

**Figure 10:**
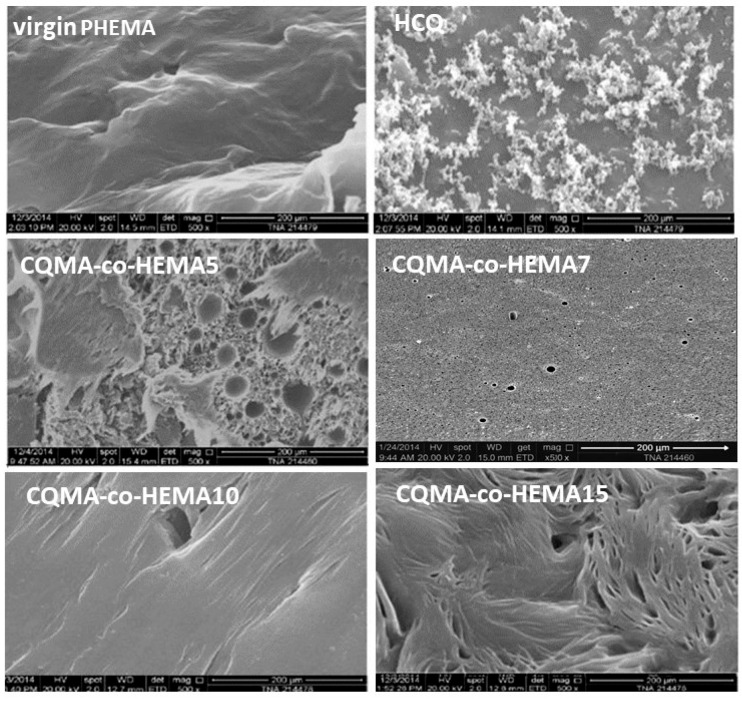
SEM micrographs of HCQ particles, surface morphologies of virgin PHEMA and CQMA-*co*-HEMA copolymers films.

Figure 10 shows the SEM surface morphology of a virgin PHEMA film, HCQ particles and CQMA-*co*-HEMA copolymer films. As can be seen in the blank PHEMA image, there was a smooth, non-porous, wave-shaped surface devoid of any aggregated residual particles of HCQ or CQMA. The image (on the right) shows the HCQ particles clustered together as endangered snowflakes, indicating the presence of moderate attractive electrostatic forces between them. Micrographs of CQMA-*co*-HEMA films showed pores of different shapes and sizes on their surfaces, which varied depending on the amount of HCQ grafted. Indeed, the CQMA-*co*-HEMA5 and CQMA-*co*-HEMA7 samples exhibited surface morphologies containing spherical pores of sizes between 3 and 70 µm, and were much denser in the case of the film sample containing 5% of CQMA by weight. The CQMA-*co*-HEMA10 image showed a smooth surface containing significantly fewer pores of similar sizes to the two previous copolymer films. The film surface of the highest HCQ-loaded copolymer sample (CQMA-*co*-HEMA15) exhibited fewer spherically shaped or compressed pores, probably due to the mechanical stress during film preparation.

### 3.3. Swelling Behavior of CQMA-co-HEMA Systems

**Figure 11:**
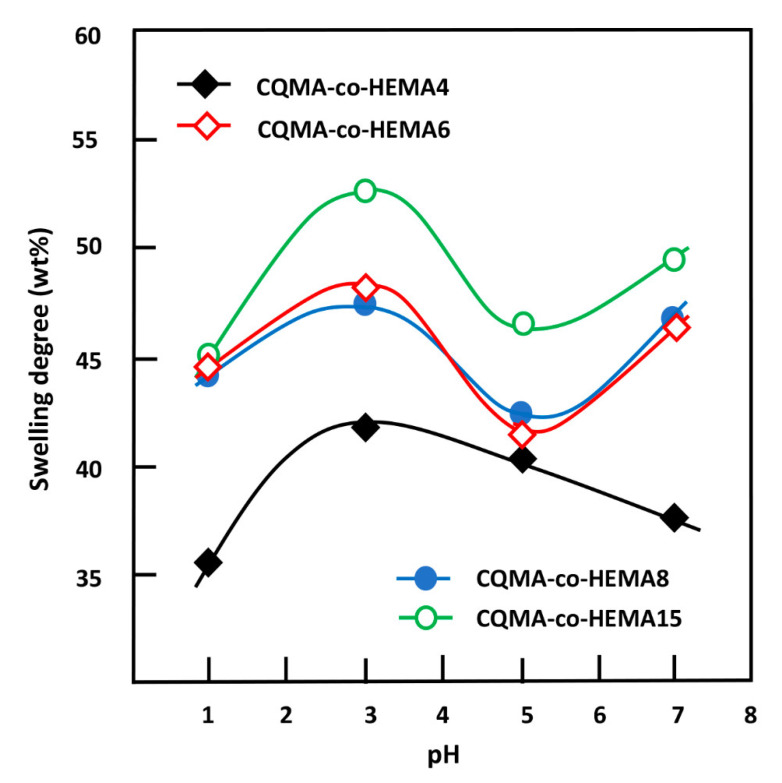
Variation of the swelling degree at equilibrium of CQMA-*co*-HEMA systems versus the media pH.

The swelling behavior of any hydrogel is an important key which controls the amount and transfer of drug released from a drug carrier system to a target external medium. The mechanism of the swelling of a polymer gel in a liquid medium mainly involves two important factors, which are the affinity of the polymer–solvent type and the diffusion dynamics of the absorbate in the absorbent material. The affinity of the polymer with respect to the absorbate is governed, mainly, by the difference between their solubility parameters. On the other hand, the diffusion dynamics of the molecules of the absorbate in the absorbent polymer matrix are controlled by various parameters such as the degree of the crosslinking of the polymer [33,34], the temperature [35,36] and the pH of the medium in which the drug is to be released [37,38]. In this work, the swelling degree at the equilibrium (S) of CQMA-*co*-HEMA films was calculated at different pH media from Equation (3) and the results obtained are plotted in Figure 11.

**Scheme 3:**
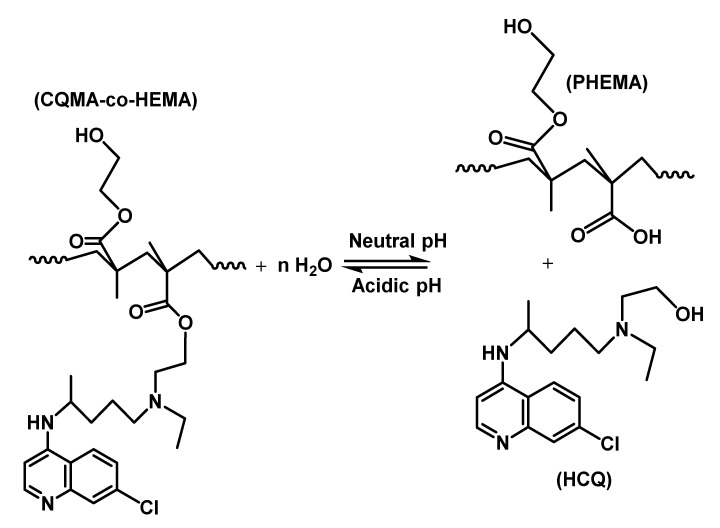
Esterification/retroesterification reactions between PHEMA and HCQ.

As can be observed from these curve profiles, the capacity of the CQMA-*co*-HEMA system to swell did not vary continuously with the pH medium, regardless of the copolymer composition. Indeed, for the drug carrier systems containing a CQMA content equal or superior to 6.22 wt%, the swelling degree at saturation reached a maximum in pH media 3 and 7, then passed by a minimum when the pH medium was 5, while for the CQMA-*co*-HEMA system containing less CQMA (4.70 wt%), the variation in the swelling rate at equilibrium reached its maximum at pH medium 3, then continuously decreased to reach a minimum at pH 7. Similar results were also obtained by different authors on the swelling capacity of PHEMA-containing amine groups [39,40]. These authors explain the increase in the swelling degree by the positive charge on the amine after protonation in the acidic medium. According to these authors, the electrostatic repulsion gives more volume to the hydrogel, allowing the diffusion of a higher water content. Similar results were also obtained by Kost et al. [40]. In this case, the greater the amount of CQMA in the copolymer, the greater the swelling capacity. This seemed to be true at a certain limit of CQMA content, but the three copolymers containing equal amounts of CQMA or more than 7.22 wt% practically had the same swelling capacities in pH medium 1. At pH 3, the swelling at equilibrium reached its maximum for all the samples. Similar results were also obtained by Sari et al. [39] in the case of copolymers involving HEMA and *N*,*N*-dimethylaminoethyl methacrylate (DMAEMA) monomers. At a high CQMA content (9.68 and 14.82 wt%), the increase in the release dynamic in the neutral pH medium was probably due to the elimination of HCQ from the copolymer through a retroesterification reaction, which was favorable in the medium at pH 7 as shown in Scheme 3. In this case, the hydrophilic contact density between hydroxyl (HEMA) and hydroxyl (water) increased at the expense of the hydrophobic contact density between carboxyl ester and hydroxyl water, resulting in a higher swelling of the system.

**Figure 12:**
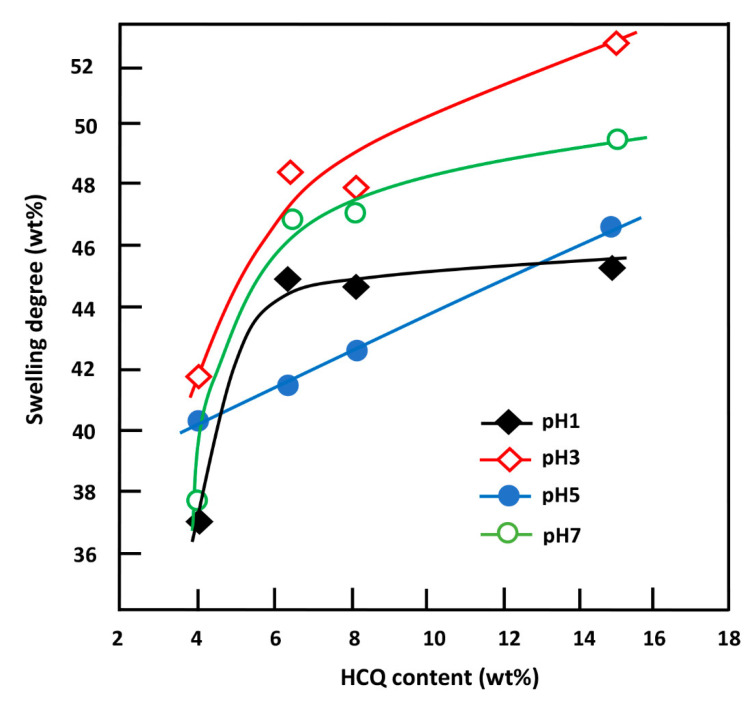
Variation of the swelling capacity of CQMA-*co*-HEMA systems in different pH media versus the HCQ content.

Figure 12 shows the change in the swelling capacity of the CQMA-*co*-HEMA copolymer at equilibrium as a function of the CQMA content. These curve profiles revealed a linear dependence of the swelling capacity for the copolymer with the CQMA content in pH medium 5 and logarithmic in pH 1, 3 and 7. As can also be seen from these data, in any pH medium investigated, the swelling rate of copolymers increased quickly when the CQMA content in the copolymer ranged between 4.70 and 7.22 wt%, while an increased linearly in pH medium 5 in all the investigated composition ranges was seen. The increase in the swelling capacity of the copolymer with the CQMA content could be explained by two main factors: (i) the increase in the solvation of the chloroquinyl methacrylate substituent through the protonation of the amine group which occurred in acidic media and (ii) the increase in the density of the hydrogen bonds between the hydroxyl of water and that of the HEMA unit through an increase in the free volume between the copolymer chains created by the bulky chloroquinyl methacrylate substituent. At a certain limit of the CQMA content, the hydrophobic character of the ester substituent intervened by limiting the solvation of the copolymer, which reflected the slowing down of the swelling capacity of copolymers containing more than 7.22 wt% in the CQMA unit, notably in pH medium 1, in which a plateau was obtained resulting from a pseudo-equilibrium between a decrease in the hydrophilicity of the copolymer due to the formation of ester (chloroquinyl methacrylate) and, simultaneously, an increase in the solvation of HCQ due to the protonation of its amine groups.

### 3.4. Cell Viability of HCQMA-co-HEMA on Ca9-22 Cells

**Figure 13:**
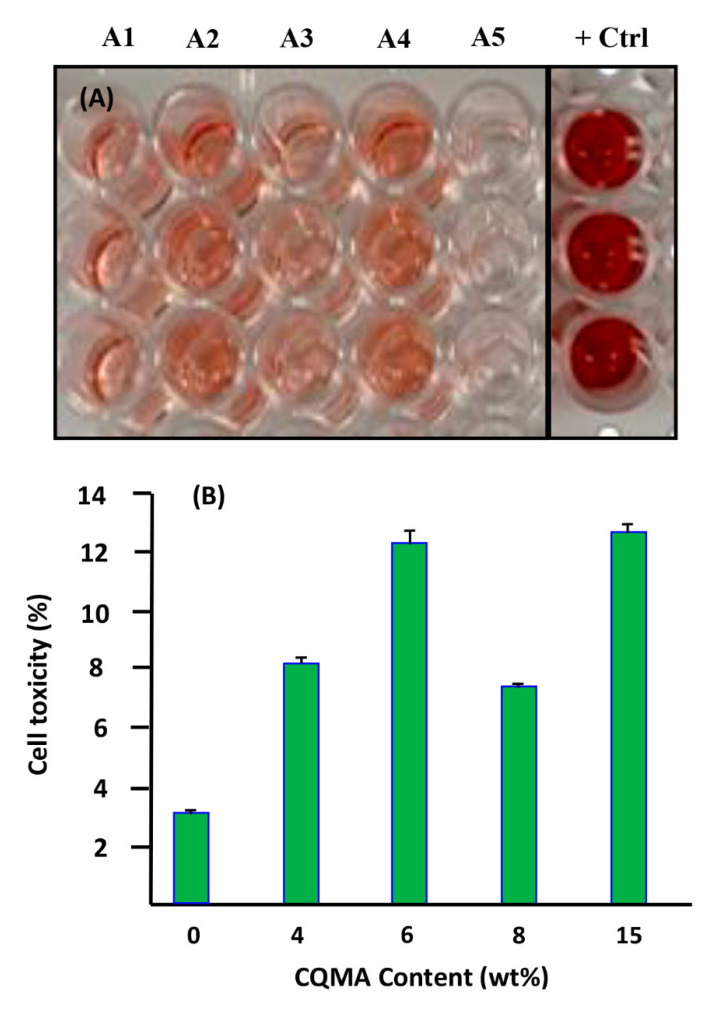
Effect of CQMA-*co*-HEMA on Ca9-22 cells cytotoxicity of different CQMA contents. (**A**) LDH assay. (**B**) Representative figure of percentage of cell toxicity with different CQMA contents.

**Figure 14:**
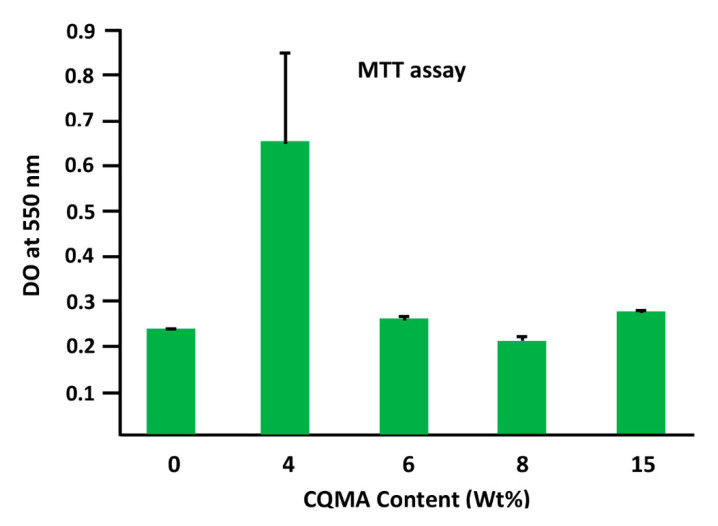
The effect of HCQ content in the CQMA-*co*-HEMA drug carrier system on Ca9-22 cell adhesion.

As shown in Figure 13, Ca9-22 cells, when treated with the polymer, presented a low toxicity between 2% and 13%. However, when the cells were treated with the drug carrier systems, an increase in cell cytotoxicity was observed for CQMA-HEMA5 (A1) (8.2 ± 0.2%), CQMA-HEMA7 (A2) (12.28 ± 0.4%), CQMA-HEMA10 (A3) (7.36 ± 0.12%), CQMA-HEMA15 (A4) (12.68 ± 0.27%), CQMA-*co*-HEMA15 (A5) and 3.07 ± 0.10% for the virgin PHEMA used as the reference. The increase in cell cytotoxicity was due to the effect of HCQ released as an anti-oral cancer agent (Figure 14).

### 3.5. Cell Adhesion and Growth

**Scheme 4:**
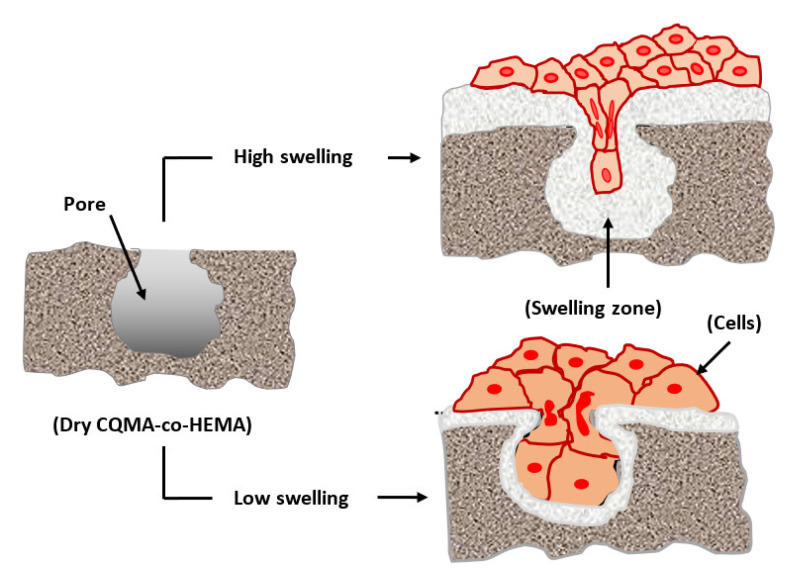
Effect of the swelling on the cell adhesion and growth.

Figure 14 shows the effect of the CQMA content in the CQMA-*co*-HEMA drug carrier system on Ca9-22 cell adhesion. As emerged from these histograms, the system containing the least CQMA (4.70 wt%) seemed to have the best performance (0.64 ± 0.19) compared to that of the reference film (virgin PHEMA) (0.23 ± 0.002). Beyond this amount grafted into the polymer, this performance almost returned again to that of the virgin polymer with a slight increase (0.27 ± 0.01) and a slight decrease (0.20 ± 0.008) when the CQMA content was 14.82 and 9.68 wt%, respectively. The adhesion of cells to a polymeric material could be managed by three essential factors which were toxicity, chemical affinity between the two target entities and surface porosity. In this case, according to the toxicity test (Figure 13), the grafting of HCQ onto PHEMA slightly affected (4.2–9.5%) the viability of the virgin material. Regarding the affinity between the polymer and the cell, this seemed to be favored by the presence of hydrogen bonds between the hydroxyl groups of the polymer and those of the cell. The size of the pores on the surface of the polymer carrier played an important role in cell adhesion and growth, because the larger the pore size, the greater the penetration of cells, the more it grows and the greater its adhesion. In this work, it was found that the best cell adhesion was observed in the system containing 4.70 wt% CQMA. This appeared to be quite consistent with the swelling results as a function of the amount of drug grafted onto the polymer (Figure 12). Because the more the swelling, the less the size of the pores as shown in Scheme 4.

### 3.6. In Vitro HCQ Release

#### 3.6.1. Release Kinetics of HCQ

**Figure 15:**
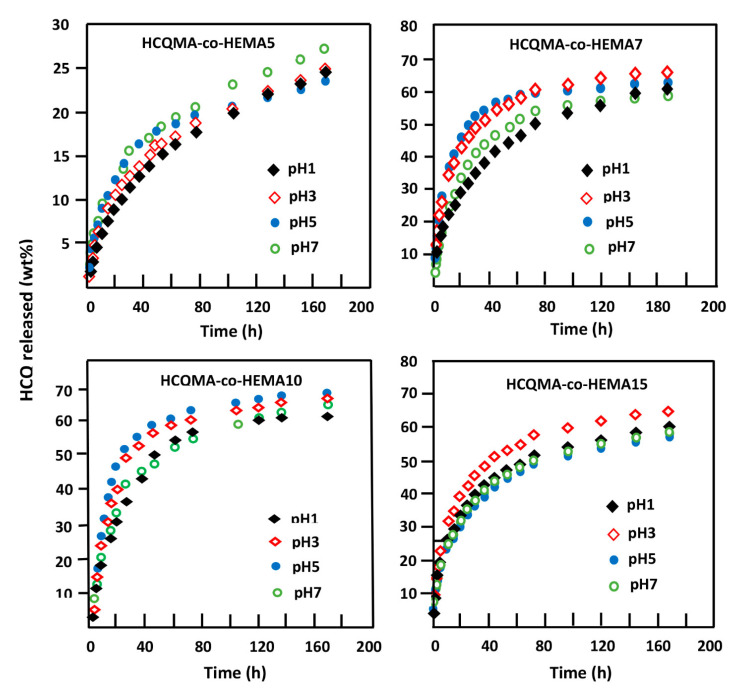
Release behavior of HCQ from CQMA-*co*-HEMA systems with different HCQ content versus time in different pH media.

Figure 15 shows the profiles of the curves indicating the change in HCQ released at 37 °C from CQMA-*co*-HEMA drug carrier systems in different pH media as a function of time over a week. As can be seen from these plots, the CQMA-*co*-HEMA10 system appeared to be the most efficient in releasing HCQ in all pH media. In fact, 62% of this drug by weight was released in pH medium 1, 68% by weight at pH medium 3, 69% by weight at pH medium 5 and 66% by weight at a neutral pH. Just after came the CQMA-*co*-HEMA7 and CQMA-*co*-HEMA15 systems, releasing during the same period 66% and 64% of 2-hydroxychloroquine by weight in pH medium 3, respectively, and 59% by weight for both in the neutral pH medium. On the other hand, CQMA-*co*-HEMA5, although it delivered much less of this drug, the maximum amount released was observed in the neutral pH medium with 27 wt% and between 24 and 25 wt% in acidic media.

#### 3.6.2. HCQ Solubility Enhancement

The solubility of any drug in an aqueous medium is a key factor in any drug delivery process, because it governs its rate and kinetics of absorption by target organs. The solubility of pure HCQ in water reported in the literature was estimated at 26.1 mg·L^−1^ at 25 °C [15]. It is well known that the solubility of a poorly water-soluble drug increases with a decrease in the size of its dispersed particles [41,42,43]. Indeed, the decrease in the size of the drug particles leads to an increase in their total surface area, leading to an increase in the contact surface water molecule drug particles. This promotes the increase in the dissolution of a very large number of particles, particularly when they are slowly released into water in the molecular state through a retroesterification reaction. In this work, the solubility of HCQ in different pH media was approximately estimated through the cumulative amount of this drug released until saturation (equilibrium) and the results obtained are given in Table 5.

**Figure 16:**
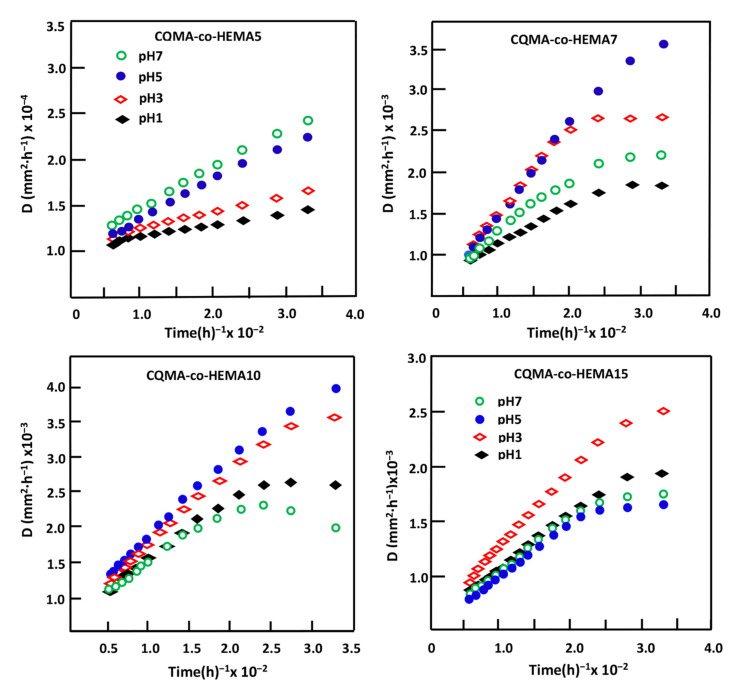
Variation of the diffusion factor of HCQ in HCQMA-*co*-HEMA systems versus reverse time.

In this work, the solubility of pure HCQ powder estimated at 37 °C in a neutral pH medium was 27.82 mg·L^−1^ and increased slowly when the pH of the medium decreased to reach 35.62 mg·L^−1^ in pH1. On the other hand, the concentrations of HCQ released in these solutions from the CQMA-*co*-HEMA drug carrier systems during 164 h were much higher, with the exception of the one containing the least HCQ (4.70% by weight), in which the concentration obtained during this same period did not exceed 22.61 mg·L^−1^, regardless of the pH of the medium. This seemed to be obvious, since 4.70 wt% as the HCQ/HEMA starting composition was not sufficient to release a sufficient amount of HCQ to reach or approach the saturation of the medium. Compared to the solubility of dissolved HCQ in powder form, this value was slightly lower. For example, the maximum concentration of HCQ released in the water of the CQMA-*co*-HEMA15 system varied between 186 and 261 mg·L^−1^ when the pH of the medium went from 1 to 7 without observing any precipitation or cloudiness for one week. This represented an improvement of over 5.22 to 9.38 times the solubility of HCQ when dissolved in the powder form. In the case of the CQMA-*co*-HEMA5 system, in which the release dynamics continued to increase (Figure 16), the lower concentrations of HCQ obtained in the different pH media obtained at the end of the release process were due to the lower amount of HCQ initially incorporated into the copolymer.

#### 3.6.3. Diffusion Behavior of HCQ

According to Lin et al. [44], for a release of less than 60 wt% from the total drug loaded, the diffusion of this substance through the carrier system follows a Fickian model. The value of the diffusion coefficient, *D*, can be calculated from Equation (4) [45,46,47,48]:

(4) $$D=\frac{0.196\times l^{2}}{t}{\left(\frac{m_{t}}{m_{o}}\right)}^{2}$$

where *l* is the film thickness, $m_{o}$ and $m_{t}\;$ are as defined previously. The *D* value is determined when the permanent regime of the release process is reached and the HCQ particles deposited on the surface of the material are totally washed. Under such conditions, the profile of the *D* curve as a function of time would be significant and accurately reflect the dynamic of the drug released into the aqueous solution inside the carrier material. Figure 16 indicates, for all samples, the variation of *D* as a function of the reverse of time plotted from the data of Figure 15 and Equation (4). The profiles of the curves obtained clearly showed two types of diffusion which occurred during the HCQ release process. The first one was rapid, which took place during the first hours of the process, mainly due to a detachment and a leaching of HCQ particles from the surface of the sample; the second, which was long, described a straight line indicating an establishment of a permanent regime. This indicated that the HCQ diffusion process through CQMA-*co*-HEMA materials obeyed a Fickian model and also indicated that the release dynamics of this drug from these hybrid materials were mainly governed by a diffusion mechanism through the copolymer matrix. Under these conditions, the steady state of the liberation process was reached and it was possible to build our investigation on the second zone of the liberation process in which the steady state was reached.

#### 3.6.4. Effect of the Swelling Degree of CQMA-*co*-HEMA Systems

**Figure 17:**
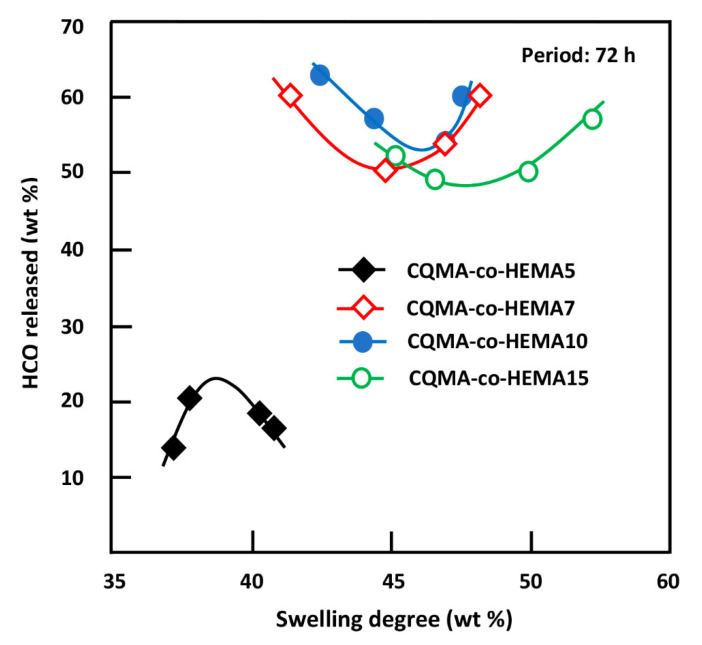
Release behavior of HCQ versus the swelling capacity of CQMA-*co*-HEMA systems in different pH media.

The swelling capacity of a polymer in an aqueous medium is a fundamental property and considered as a key in the field of drug delivery. Indeed, this parameter regulates the amount of drug released, controls both the rate of diffusion of the penetrate into the polymeric carrier and its dissolution in the medium in which the drug is released [49,50,51,52]. In this work, the influence of the swelling capacity of the CQMA-*co*-HEMA system on the release behavior of hydroxychloroquine was carried out in different pH media during 72 h of the release process and the results obtained are plotted in Figure 17. As can be observed from these curve profiles, minimum releases of 53.52, 50.02 and 48.32 wt% were obtained for the drug carrier systems containing 9.68, 7.22 and 14.82 wt% of HCQ content, respectively, whereas for the system containing the lowest HCQ content (4.70% by weight), the dynamics behaved differently, which passed by a maximum of 23.70 wt% at a swelling saturation of 38.70% by weight.

#### 3.6.5. Effect of the CQMA Content

**Figure 18:**
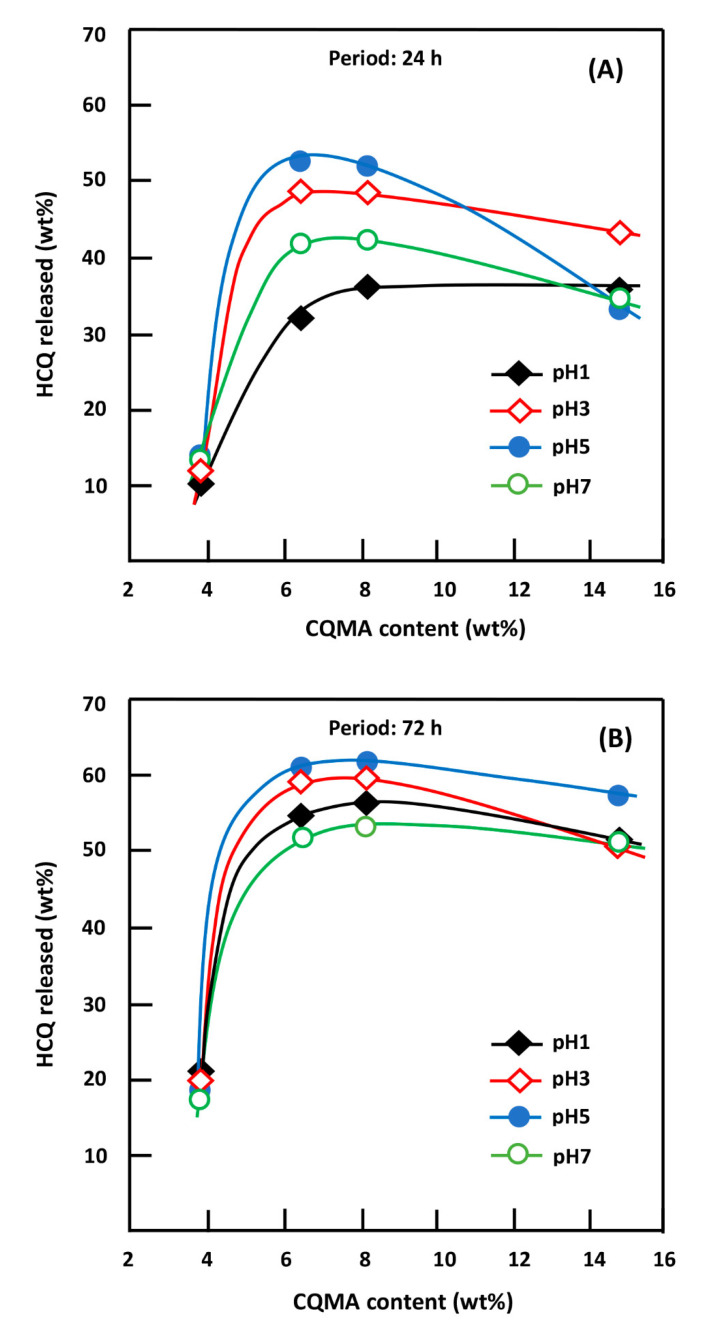
Variation of the HCQ released during 24 h (**A**) and 72 h (**B**) of the release process versus the CQMA content in the drug carrier system and in different media pHs.

Figure 18 shows the influence of the CQMA content in the CQMA-*co*-HEMA drug carrier system on the release dynamics during 24 and 72 h of the process. For both durations, these curve profiles revealed a significant increase in the HCQ released when the concentration of this medication in the drug carrier system did not exceed 7.22% by weight, then stabilized or decreased more or less quickly depending on the pH medium at a higher HCQ content. This could be explained mainly by two opposing factors which acted simultaneously on the swelling capacity of the drug vehicle system depending on their capacities to act and the content of CQMA in the copolymer. In fact, an increase in the hydroxychloroquinyl ester group as a substituent of the copolymer acted negatively on the hydrophilic power of the CQMA-*co*-HEMA system. This had an effect of reducing the quantity of water necessary for the reaction of retroesterification (drug regeneration reaction) and also reduced the dissolution of HCQ released inside the polymeric matrix.

On the other hand, an increase in the steric hindrance of this substituent also promoted an increase in the free volume between the chains of the copolymer, facilitating the penetration of more water molecules. In this situation, a competition between these two opposite effects, which essentially depends on the amount of HCQ grafted into the copolymer, governs the dynamics of HCQ release in the medium. Finally, we could conclude from the results obtained that the ability to release HCQ in these different media was governed by the more or less hydrophilic character of the CQMA-*co*-HEMA system, which depended on its CCQMA/PHEMA composition.

#### 3.6.6. Effect of pH Media

**Figure 19:**
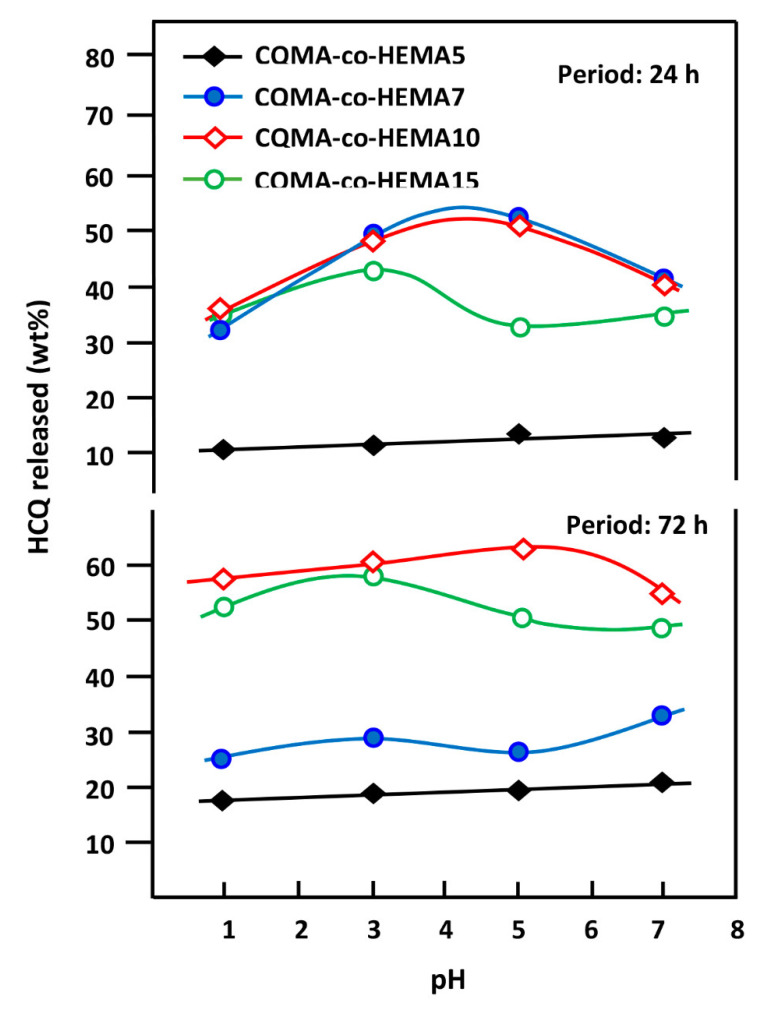
Variation of the cumulative HCQ released from the CQMA-*co*-HEMA drug carrier systems versus the media pH.

The nature of the environment in which drugs are released greatly influences the dynamics of drug release in a target organ. Indeed, various studies have been carried out on the influence of the medium such as the pH [53,54], the enzymes [54,55] and the bacteria [56,57] on the dynamics of the drug released by the drug delivery systems and the results obtained were very striking. In this work, the effect of pH media on the dynamics of HCQ released from CQMA-*co*-HEMA drug carrier systems was performed at pH media 1, 3, 5 and 7, and the results obtained for 24 and 72 h are plotted for comparison in Figure 19. As can be seen from the profiles of the curves obtained, for the drug carrier system containing the least CQMA (4.70% by weight), virtually no change in the release dynamics was observed, regardless of the pH of the medium. At 24 h of the release process, the two systems containing 7.22 and 9.68 wt% of CQMA content evolved according to the same trend, in which the release dynamics passed by a maximum of 54 wt% for the first system and 52 wt% for the second when the pH of the medium was 4.2. In contrast, for drug carrier systems containing 4.70 and 14.82% of CQMA by weight, the release dynamic continued to decrease slightly and linearly for the first drug carrier system to reach 10.35 wt% of HCQ in pH medium 7, while that of the second system passed through an inflection point at 37% of HCQ by weight released in pH medium 4.2. At 72 h of release process, the dynamics of HCQ released from the CQMA-*co*-HEMA7 and CQMA-*co*-HEMA10 drug carrier systems, which contained 7.22 and 9.68 wt% of CQMA, respectively, completely changed from those observed at 24 h. Indeed, two extremums were observed for the drug carrier system containing 7.22 wt% of CQMA content in which a maximum release of 29.7 wt% of HCQ was observed in Ph 3 and a minimum of 26.6 wt% in pH medium 5, while that with 9.68 wt% of CQMA content reached a maximum release of 63 wt% in this same pH medium. The explanation for such behavior appeared to be complicated by the fact that two main antagonistic factors may simultaneously intervene in the drug release mechanism: the first being chemical which resides in the reaction of retro-esterification which generates the HCQ and the second being physical which resides in the hydrophilic character of the copolymer. The intensity of each depends on the pH of the medium and the CQMA content in the drug carrier system. Indeed, the chemical factors were the reactions of retro-esterification which took place in a neutral pH medium which regenerated the HCQs in the presence of an excess of water, leading to the release of a large amount of drug, and the reaction of esterification which took place in an acidic medium, which, on the contrary, promoted the formation of insoluble ester, which disfavored the release of the drug as shown in Scheme 3. The physical factor that could affect the release dynamic of HCQ from the CQMA-*co*-HEMA system was already discussed in the previous section. Indeed, at a high CQMA content, the swelling capacity of the polymer was also affected by the pH medium, since the more acidic the medium the less swelling of the system. This was due to the formation of the ester which was insoluble in water. Contrarily, as can be revealed in the previous section, a minimal amount of CQMA in the copolymer, less than 7.22% by weight, promoted an increase in the swelling capacity.

#### 3.6.7. Performance of CQMA-*co*-HEMA Drug Carrier System

The study of the performance of the CQMA-*co*-HEMA drug carrier system based on the drug amount released the instantaneous release rate and the duration of the release process, deduced from the slopes of the pseudo-linear portions of the kinetic curves of Figure 15, led to the results of Table 6.

As can be seen from these curve profiles, the release dynamic of HCQ showed two principal pseudo stable zones for the specimen containing 4.70 wt% of CQMA and a supplementary transit zone for the CQMA-*co*-HEMA drug carrier systems with a higher CQMA content. The first zone observed during the first hours of the release process was between 10 and 30 h depending on the composition of the copolymer and the pH of the medium. During this period, a significant amount of HCQ was released, probably due to the large difference between the chemical potential for dissolving HCQ outside and inside the polymer matrix. For the CQMA-*co*-HEMA5 drug carrier system, a rapid release dynamic (0.43–0.53 wt%/h) was observed during the second zone which was located between 120 and 134 h in which 6.0 to 18.2% of HCQ by weight was released depending on the pH medium. For drug carrier systems initially containing a higher CQMA content, the second zone was short (32–43 h) and relatively fast (0.10–0.63 wt%/h). This step was considered as a transit zone in which 9.0–22.0% of drug by weight was released during this period depending on the CQMA content in the copolymer and the pH of the medium. The third zone observed for CQMA-*co*-HEMA drug carrier systems containing 4.70, 9.68 and 14.82 wt% CQMA was the longest (91–116 h) and also the slowest (0, 06–0.13 wt%/h), wherein 7.0–13.0 wt% HCQ was released depending on the initial content of CQMA in the drug carrier system and the pH medium. The gradual decrease in the release dynamics of these drug delivery systems can mainly be explained thermodynamically by a gradual decrease in the difference between the chemical potentials of the HCQ dissolution inside and outside the polymer matrix up until the equilibrium is reached, taking into account the various parameters of interaction between the various components of the copolymer and that of the medium. The performance of the drug carrier systems was determined from the criteria that stipulate: the maximum of drug released in neutral pH medium (intestinal transit), minimum drug release in acidic pH medium (stomach), stable release rate and longest release period. According to Belzer et al. [58], the mean total gastrointestinal transit time (GITT) was between 53 and 88 h distributed over three main stages: (i) stomach transit (pH ≈ 1.5–3.5), which lasts between one and 4 h, (ii) intestinal transit (pH ≈ 7–9), which varies between 4 and 12 h and, finally, (iii) transit in the colon (pH ≈ 5–7), which lasts between 48 and 72 h. Taking into account the pH of the medium and the transit times in different digestive organs, it was possible to approximately estimate from the data of Table 7 the distribution of the percentages of cumulative HCQ released in different organs and the average stomach/digestive organs rate (SDO) (Equation (5)), independently of the effects of enzymes and microorganisms and the results obtained are grouped in Table 7. These data revealed that the CQMA-*co*-HEMA5 system was the most efficient, because it was able to reduce the release of HCQ in the stomach to 2.40 wt% of the total quantity released for the fast GITTs and 6.69 wt% for the slow GITTs.

(5) $$SDO\left(\mathrm{wt}\%\right)=\frac{r_{s}}{r_{s}+r_{si}+r_{c}}\times 100,$$

where $r_{s}$ , $r_{si}$ and $r_{c}$ are the percentages of HCQ released in the stomach, small intestine and colon, respectively, during a certain transit time.

## 4. Conclusions

Very interesting results were obtained from this investigation. Indeed, 2-chloroquinyl methacrylate as a new monomer could be easily synthesized through a catalytic reaction involving methacryloyl chloride in the presence of triethylenetetramine. Poly(2-chloroquinyl methacrylate-*co*-2-hydroxyethyl methacrylate drug carrier systems with different compositions were also successfully synthesized through a radical copolymerization route involving the synthesized 2-chloroquinyl methacrylate and 2-hydroxyethylmethacrylate. This method was able to obtain, in aqueous medium, a generator of a desired percentage of 2-hydroxychloroquine regularly and slowly released during a long period through a retro-esterification reaction. The cell toxicity examined by the LDH assay revealed that the grafting of HCQ onto PHEMA was slightly affected (4.2–9.5%) and the cell adhesion and growth on the CQMA-*co*-HEMA drug carrier specimens carried out by the MTT assay revealed the best performance with the specimen containing 4.70 wt% CQMA. A significant improvement of 5.22 to 9.38 times the solubility of HCQ over that in powder form was obtained when it was released from CQMA-*co*-HEMA systems. The results of the in vitro release dynamics of HCQ from these systems showed very encouraging results in which the system initially containing 4.70% of CQMA by weight showed the best performance.

## Figures and Tables

**Table 1:** Some physicochemical characteristics of 2-hydroxychloroquine [24].

Formula	Molar Mass (g∙mol^−^^1^)	Appearance	Density (g∙L^−^^1^)	Water Solubility (mg∙L^−^^1^)	Melting Temperature (°C)
C_18_H_26_ClN_3_O	335.87	White crystals	1.2	26	90

**Table 2:** Experimental conditions of the synthesis of CQMA-*co*-HEMA copolymer with different CQMA content.

Copolymer	CQMA (g)	HEMA (g)	AIBN (wt%)	Monomers Composition (wt%)	Drug Content (wt%)
CQMA-*co*-HEMA5	0.20	9.80	0.1	10	2.0
CQMA-*co*-HEMA7	0.85	9.15	0.1	25	8.5
CQMA-*co*-HEMA10	1.00	9.00	0.1	50	10.0
CQMA-*co*-HEMA15	1.50	8.50	0.1	75	15.0

**Table 3:** Comparison between the experimental determination of the elementary composition of the CQMA and that calculated.

Element	Composition (wt%)					
	Sulfanilamid (Ref)		HCQ		CQMA	
	Exp.	Calc.	Exp.	Calc.	Exp.	Calc.
C	41.850	41.860	64.230	64.310	65.470	65.367
H	04.680	04.651	07.787	07.741	07.489	07.428
N	16.260	16.279	12.393	12.505	10.450	10.399

**Table 4:** Composition of CQMA-*co*-HEMA determined by CHN elementary analysis.

System	Elementary Composition (wt%)			CQMA (wt%)	HCQ (mol%)
	C	H	N		
PHEMA	55.287	7.723	0	0	0
CQMA-*co*-HEMA5	56.747	7.602	01.412	04.70	1.61
CQMA-*co*-HEMA7	59.906	5.294	02.116	07.22	2.52
CQMA-*co*-HEMA10	59.496	7.720	02.673	09.68	3.44
CQMA-*co*-HEMA15	57.110	7.673	01.852	14.82	6.49
PCQMA	65.362	7.540	10.563	100	100

**Table 5:** Solubility of HCQ in different pH media at 37 °C deducted from the maximum amount of HCQ released from CQMA-*co*-HEMA drug carrier systems.

Compound	Solubility (mg∙L^−^^1^)			
pH	1	3	5	7
HCQ powder	35.62	31.17	28.08	27.82
CQMA-*co*-HEMA5	22.61	21.41	19.0	19.32
CQMA-*co*-HEMA7	168.0	149.0	137.0	167.0
CQMA-*co*-HEMA10	58.0	149.0	70.0	84.0
CQMA-*co*-HEMA15	186.0	249.0	252.0	261.0

**Table 6:** Performance of the CQMA-*co*-HEMA drug carrier systems.

System	pH	Stable Zone (h)	HCQ Released (wt%)	Release Rate (wt%/h)	System	Stable Zone (h)	HCQ Released (wt%)	Release Rate (wt%/h)
CQMA-*co*-HEMA5	1	0–27 30–164	12.0 ± 0.2 12 ± 0.5	0.44 ± 0.04 0.10 ± 0.03	CQMA-*co*-HEMA7	0–15 15–62 62–164	22.0 ± 0.8 22.0 ± 0.7 10.0 ± 0.3	1.46 ± 0.02 0.47 ± 0.02 010 ± 0.02
	3	0–30 44–164	13 ± 0.2 18.2 ± 0.5	0.43 ± 0.04 0.15 ± 0.01		0–10 10–60 60–164	26.03 ± 0.6 18.00 ± 0.6 10.0 ± 0.6	2.60 ± 0.04 0.36 ± 0.02 0.10 ± 0.06
	5	0–37 50–164	17.0 ± 0.6 6.0 ± 0.7	0.46 ± 0.08 0.05 ± 0.04		0–10 10–60 60–164	37.0 ± 0.6 17.0 ± 0.5 13.0 ± 0.6	3.70 ± 0.07 0.34 ± 0.03 0.13 ± 0.06
	7	0–30 30–164	16.0 ± 0.5 12.0 ± 0.8	0.53 ± 0.04 0.09 ± 0.03		0–22 22–65 65–164	38.0 ± 0.4 18.0 ± 0.5 7.0 ± 0.6	1.73 ± 0.10 0.42 ± 0.02 0.07 ± 0.06
CQMA-*co*-HEMA10	1	0–20 20–58 58–164	30.0 ± 0.3 22.0 ± 0.4 7.0 ± 0.4	1.5 ± 0.08 0.58 ± 0.09 0.070 ± 0.04	CQMA-*co*-HEMA15	0–10 10–48 48–164	26.0 ± 0.3 20.0 ± 0.3 13.0 ± 0.02	2.60 ± 0.04 0.53 ± 0.05 0.11 ± 0.04
	3	0–20 20–60 60–164	40.0 ± 0.3 9.0 ± 0.5 9.0 ± 0.4	2.3 ± 0.02 0.23 ± 0.02 0.09 ± 0.04		0–10 10–42 50–146	31 ± 0.4 20 ± 0.4 10.0 ± 0.4	3.10 ± 0.06 0.63 ± 0.04 0.06 ± 0.04
	5	0–20 20–60 60–164	46.0 ± 0.3 14.0 ± 0.4 9.0 ± 0.4	2.30 ± 0.02 0.35 ± 0.09 0.09 ± 0.04		0–12 12–56 56–164	24.0 ± 0.5 22.0 ± 0.5 10.0 ± 0.04	2.00 ± 0.06 0.50 ± 0.05 0.09 ± 0.04
	7	0–28 28–73 73–164	41.0 ± 0.4 13.0 ± 0.5 10.0 ± 0.4	1.46 ± 0.06 0.29 ± 0.02 0.11 ± 0.04		0–12 12–54 54–164	25.0 ± 0.5 21.0 ± 0.5 10.0 ± 0.04	1.93 ± 0.04 0.10 ± 0.05 0.09 ± 0.4

**Table 7:** Estimated distribution of the cumulative HCQ released from CQMA-*co*-HEMA drug carrier systems on the principal digestive organs timed according to Belzer.

CQMA-*co*-HEMA System	Stomach Transit (wt%)		Small Intestine Transit (wt%)		Colon Transit (wt%)		SDO (wt%)	
Transit Time	Min (1 h)	Max (4 h)	Min (4 h)	Max (12 h)	Min (48 h)	Max (72 h)	Min (48 h)	Max (72 h)
CQMA-*co*-HEMA5	0.44	1.74	2.12	6.36	15.76	17.90	2.40	6.69
CQMA-*co*-HEMA7	2.03	8.12	6.92	20.76	43.68	49.86	3.86	10.31
CQMA-*co*-HEMA10	1.90	7.60	5.84	17.52	40.96	47.92	3.90	10.40
CQMA-*co*-HEMA15	2.85	11.4	7.72	23.16	20.98	23.38	9.03	19.68

## Data Availability

The data presented in this study are available on request from the corresponding author.
