# Correction: Aljubailah et al. Copolymer Involving 2-Hydroxyethyl Methacrylate and 2-Chloroquinyl Methacrylate: Synthesis, Characterization and In Vitro 2-Hydroxychloroquine Delivery Application. *Polymers* 2021, *13*, 4072

**DOI:** 10.3390/polym18091083

**Published:** 2026-04-29

**Authors:** Abeer Aljubailah, Wafa Nazzal Odis Alharbi, Ahmed S. Haidyrah, Tahani Saad Al-Garni, Waseem Sharaf Saeed, Abdelhabib Semlali, Saad M. S. Alqahtani, Ahmad Abdulaziz Al-Owais, Abdulnasser Mahmoud Karami, Taieb Aouak

**Affiliations:** 1 Department of Chemistry, College of Science, Imam Mohammad Ibn Saud Islamic University (IMSIU), Riyadh 13623, Saudi Arabia; akaljubailah@imamu.edu.sa (A.A.); wnalharbi@imamu.edu.sa (W.N.O.A.); 2 Nuclear and Radiological Control Unit, King Abdulaziz City for Science and Technology (KACST), Riyadh 11442, Saudi Arabia; ahydrah@kacst.edu.sa; 3 Chemistry Department, College of Science, King Saud University, Riyadh 11451, Saudi Arabia; tahanis@ksu.edu.sa (T.S.A.-G.); salqahtani2@ksu.edu.sa (S.M.S.A.); aowais@ksu.edu.sa (A.A.A.-O.); akarami@ksu.edu.sa (A.M.K.); 4 Engineer Abdullah Bugshan Research Chair for Dental and Oral Rehabilitation, College of Dentistry, King Saud University, Riyadh 11545, Saudi Arabia; 5 Groupe de Recherche en Écologie Buccale, Faculté de Médecin Dentaire, Université Laval, Quebec City, QC G1V 0A6, Canada; abdelhabib.semlali@greb.ulaval.ca

## Error in Figure

In the original publication [1], there was a mistake in Figure 10 as published. The SEM image labeled CQMA-co-HEMA7 was inserted incorrectly due to an unintentional mix-up during figure compilation, caused by the large number of samples and the time elapsed since the experimental work.

The corrected Figure 10 appears below, where the SEM image of CQMA-co-HEMA7 has been replaced with the correct micrograph obtained under the same experimental conditions. The authors state that the scientific conclusions are unaffected. This correction was approved by the Academic Editor. The original publication has also been updated.

## Figures and Tables

**Figure 10:**
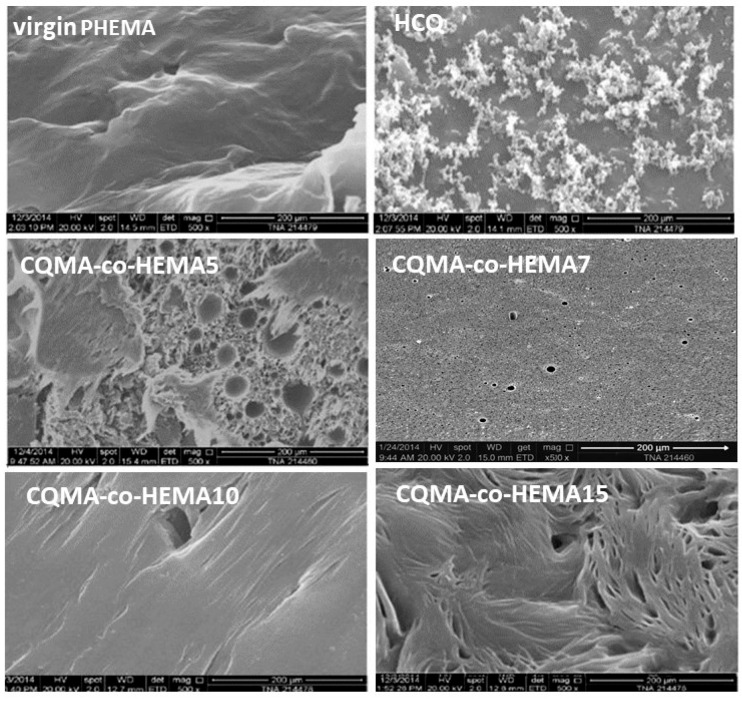
SEM micrographs of HCQ particles, surface morphologies of virgin PHEMA and CQMA-*co*-HEMA copolymers films.
